# Prevalence and antibiotic resistance profile of *Staphylococcus pseudintermedius* in pyodermic dogs in Asia: a systematic review and meta-analysis

**DOI:** 10.1007/s11259-026-11231-2

**Published:** 2026-05-01

**Authors:** Mohammad Farzad Afshar, Nasir Ahmad Sarwary, Syafiqah Ishak, Tahera Hashimi, Priscilla Nasthalina Jabing, Mohammed Babatunde Sadiq, Chen Hui Cheng

**Affiliations:** 1 Department of Veterinary Laboratory Diagnosis, Faculty of Veterinary Medicine, Universiti Putra Malaysia, Serdang, 43400 Selangor Malaysia; 2 Department of Food Technology and Hygiene, Faculty of Veterinary Sciences, Kabul University, Kabul, Afghanistan; 3 Department of Biomedical Sciences, College of Biomedicine, City University of Hong Kong, Tat Chee Ave, Kowloon, Hong Kong SAR; 4 Department of Veterinary Preclinical Sciences, Faculty of Veterinary Medicine, Universiti Putra Malaysia, Serdang, 43400 Selangor Malaysia; 5 Department of Farm and Exotic Animal Medicine and Surgery, Faculty of Veterinary Medicine, Universiti Putra Malaysia, Serdang, 43400 Selangor Malaysia; 6 Department of Veterinary Clinical Studies, Faculty of Veterinary Medicine, Universiti Putra Malaysia, Serdang, 43400 Selangor Malaysia

**Keywords:** Disk diffusion test, Methicillin-resistance, Pet, Public health, Staphylococci, *Antimicrobial resistance*

## Abstract

*Staphylococcus pseudintermedius* is the primary bacterial agent associated with canine pyoderma, with rising concern over methicillin-resistant strains in Asia. This systematic review and meta-analysis aimed to determine the prevalence of methicillin-susceptible (MSSP) and methicillin-resistant (MRSP) *S. pseudintermedius* from canine skin infections and to evaluate their antimicrobial resistance profiles. Following PRISMA (Preferred Reporting Items for Systematic Review and Meta-Analysis) guidelines, studies published between 2015 and 2024 were retrieved from Scopus, PubMed, Web of Science, and Google. Only cross-sectional studies from Asian countries reporting disk diffusion susceptibility testing in dogs with pyoderma were included. Fifteen studies met the inclusion criteria, with overall moderate methodological quality. Fourteen studies were included in prevalence analyses. The pooled prevalence of MSSP was 27.9% (95% CI: 18.8–39.3) and MRSP was 30.7% (95% CI: 21.3–42.1), with high heterogeneity between the included studies (I^2^ = 93.85%; *p* < 0.001). The pooled prevalence of the *mec*A gene among MRSP was 11.9%. Meta-regression revealed no significant difference between MSSP and MRSP prevalence (*p* = 0.853). MSSP isolates had the lowest resistance to amoxicillin–clavulanic acid and oxacillin, whereas MRSP exhibited broader resistance, particularly to oxacillin, clindamycin, and doxycycline. These findings highlight the substantial burden of MRSP in canine pyoderma in Asia and reinforce the need for susceptibility-guided therapy and strengthened antimicrobial stewardship.

## Introduction

Dogs are the most kept animals as pets (Harris 2023). Pet ownership is widespread across Asia, with millions of dogs and cats kept in countries such as Thailand, the Philippines, Taiwan, and Japan, where companion animals also contribute to mental health support through animal-assisted therapy (Rojekittikhun et al. 2014; Rakuten Insight Global 2018; Kurita et al. 2019).

*Staphylococcus pseudintermedius* is recognized as a common skin and ear pathogen of dogs and cats; in addition, it has a zoonotic risk for human beings and can cause nosocomial infections like sinusitis, soft tissue infection, and endocarditis (Bush and Bradford 2020; Guardabassi et al. 2013; Schwarz et al. 2018). *S. pseudintermedius* usually colonizes the mucocutaneous parts of healthy dogs, such as the nares, anus, groin, mouth and forehead (Papadogiannakis et al. 2016). It is also uncommonly capable of colonizing human skin, while people who have more contact with pet dogs can be carriers of this microorganism (Solanki et al. 2023; Somayaji et al. 2016; Weese and Van Duijkeren 2010). Several studies in Turkey (Metiner et al. 2015), India (AnandaChitra et al. 2016), Korea (Lee et al. 2018), Taiwan (Lai et al. 2022), China (Wang et al. 2022) and Iran (Naziri and Majlesi 2023) have reported the occurrence of *S. pseudintermedius* in their countries, with some reports of methicillin resistance among isolates and detection of isolates from the owners.

Antimicrobial resistance among Staphylococci, which are considered common commensals of the gastrointestinal tract, mucosa, and skin, is a major concern of public health; such bacteria are opportunistic pathogens as well (Rich and Rich 2005). Methicillin-resistant *Staphylococcus pseudintermedius* is an example of such bacteria (Perreten et al. 2010). Resistance to all beta-lactam antibiotics is the result of *mec*A carriage in Staphylococci (Tirosh-Levy et al. 2015). Dissemination of antimicrobial-resistant bacteria in veterinary hospitals can cause nosocomial infections; thus, increments in morbidity, mortality, and treatment costs are the results of resistant bacteria (Espadale et al. 2018).

Although there are studies on the presence and antimicrobial susceptibility pattern of *Staphylococcus pseudintermedius* in dogs in Asia (AnandaChitra et al. 2016; Jantorn et al. 2021; Lai et al. 2022; Lee et al. 2018; Metiner et al. 2015; Naziri and Majlesi 2023), there is no general antimicrobial profile pattern to help clinicians opt for an effective antimicrobial agent. Thus, the objective of this review is to determine the prevalence and resistance profile of MSSP (methicillin-susceptible *Staphylococcus pseudintermedius*) and MRSP (methicillin-resistant *Staphylococcus pseudintermedius*) isolated from canine pyoderma in different Asian countries. The findings will assist in selecting appropriate antimicrobial agents when treating *S. pseudintermedius* infections, making informed antimicrobial utilization decisions, and taking measures to reduce their resistance against various antimicrobial agents.

## Materials and methods

### Study design

This review was based on studies that reported the antimicrobial susceptibility testing of *S. pseudintermedius* using the disk diffusion method in Asian countries. The study used the guidelines for the Preferred Reporting Items for Systematic Review and Meta-Analysis (PRISMA) (Moher et al. 2015).

### Search strategy

A literature search was conducted using Scopus, PubMed, Web of Science and Google databases for research articles with a focus on the antimicrobial susceptibility of *S. pseudintermedius* in dogs with the terms “dog or canine or pet dogs, or dogs”, and “pyoderma or dermatitis or skin infection”, and “antibiotic or susceptibility and testing or susceptibility and testing”, and “*S. pseudintermedius*” or “*Staphylococcus pseudintermedius*” or “MRSP” OR “methicillin-resistant *Staphylococcus pseudintermedius*” OR “Methicillin-resistant *S. pseudintermedius*” or “*Staphylococcus intermedius* group” or ”SIG”.

### Eligibility criteria

The initial set of eligible articles was independently selected by two researchers (MFA and CHC). This systematic review included original cross-sectional studies; filters were set to include articles from Asian countries and published in bibliographic databases from 2015 to 2024. Only studies that provided sufficient information on the antimicrobial susceptibility testing of *S. pseudintermedius* using the disk diffusion method were included. As for exclusion criteria, other study designs, other species than dogs, studies conducted using other antimicrobial susceptibility testing (including minimum inhibitory concentration or VITEK), that contained duplicate data or overlapping articles, reviews, conference abstracts or proceedings, short communications or short reports were excluded. Data obtained from minimum inhibitory concentration (MIC) testing and VITEK systems were excluded to ensure methodological consistency, as only studies employing standardized disk diffusion methods were included, thereby reducing variability in susceptibility interpretation across different testing platforms. Furthermore, access to VITEK systems is not universal across diagnostic laboratories, and MIC testing is a quantitative and relatively costly approach compared with the disk diffusion method, which is qualitative and more economical. The MSSP or MRSP status was primarily defined based on phenotypic antimicrobial susceptibility testing using the oxacillin disk diffusion method, in accordance with established interpretive criteria. Studies that additionally reported *mec*A gene detection by polymerase chain reaction (PCR) were noted; however, phenotypic resistance was considered the primary criterion for classification to ensure consistency across included studies.

### Data extraction

A standardized data extraction form was developed to systematically extract relevant information. Data items extracted were first author, country and city of study, year of publication, study design, study period, source of sample, detection method, antimicrobial susceptibility testing method, total number of isolates, methicillin resistance or susceptibility, resistance genes, and susceptibility rate of *S. pseudintermedius* to specific antimicrobial agents, and cut-off value reference. After searching the databases, all citations found were exported to Mendeley before being arranged in an Excel sheet for independent and blind selection by two authors (MFA and HCC). The selection of publications was a two-step process; initially, titles and abstracts of the studies found were checked using inclusion and exclusion criteria. The full text of all eligible studies was evaluated by two authors (MFA and HCC) independently for inclusion. Any disagreement was resolved through discussion.

### Statistical analysis

Pooled prevalence estimates of MSSP and MRSP were computed using a random-effects meta-analysis of individual study prevalences. In addition, Meta-regression analysis was performed in CMA (Comprehensive Meta-Analysis software version 2.2 [2011]) using a random-effects model to compare the pooled prevalence of MSSP and MRSP, with bacterial type entered as a binary moderator. Logit-transformed event rates and method-of-moments estimation were applied, and significance was determined from the moderator *p*-value.

The prevalence of antimicrobial-resistant *S. pseudintermedius* or resistant gene carrier *S. pseudintermedius* was calculated as the number of antimicrobial-resistant isolates over the total number of isolates. The statistical analysis was performed using CMA. Given that the outcome variable is binary (i.e., *S. pseudintermedius resistant*/sensitive to the antimicrobial or carries a specific resistance gene/ or does not carry) and is given only for single groups, the only possible parameter to measure effect size is the raw proportion *p* (with 95% confidence intervals [CIs]) using a random effects model (Borenstein et al. 2009).

Study heterogeneity was assessed using Der Simonian and Laird tests (Q statistic). The inconsistency index (I^2^-statistic) was used to quantify the degree of heterogeneity (Borenstein et al. 2009). Publication bias was investigated using funnel plots, including an adjusted rank correlation test using the Egger method (Egger et al. 1997), and the Begg’s test (Begg and Mazumdar 1994).

### Risk of bias and quality assessment of the included studies

We used the Newcastle‒Ottawa Scale (NOS) to evaluate the quality and integrity of the included studies (Supplementary file Research checklist). NOS is a scoring system in which each study is rated with a point according to its selection, comparability, exposure, or outcome. The study was given a maximum of one (1) point for each of the numbered items belonging to the selection and exposure section. For the comparability domain, a maximum of two (2) points was awarded. The scores may be between 0 and 9 (Wells et al. 2011). A study was regarded as high quality if its overall quality score was seven (7) points or higher. Two authors (with initials MFA and HCC) solely evaluated the quality of the individual studies, and any discrepancies in the assessment were discussed with other authors.

## Results

### Characteristics of included and excluded articles and quality assessment

**Fig. 1:**
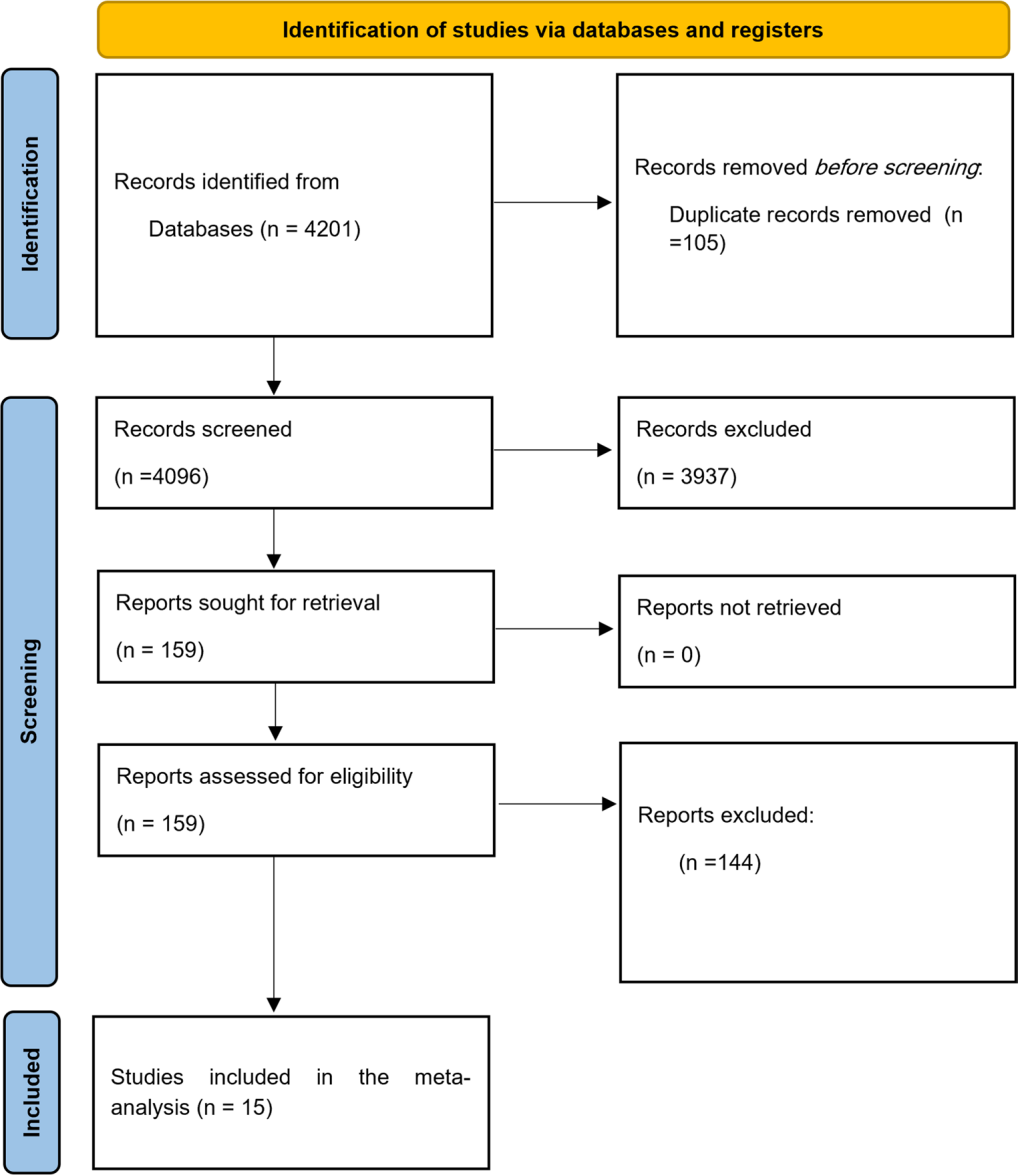
Flow diagram of included and excluded studies in the MSSP and MRSP meta-analysis

One hundred and fifty-nine articles were identified as possible sources of information at the end of the search period. Some studies were excluded because they lacked sufficient data to estimate the prevalence of *S. pseudintermedius* isolates resistant to specific antimicrobial agents within the isolate population. Other exclusion criteria were: articles from other animal species or those where *S. pseudintermedius* was not identified to the species level, review articles, articles that used other antimicrobial susceptibility testing methods, experimental studies, not differentiating the sample source (for instance, oral samples, nasal samples, rectal samples) when reporting the resistance rates, not differentiating methicillin-resistant and methicillin-susceptible when reporting resistance rates, and studies where targeted apparently healthy dogs, or did not collect samples from skin infection of dogs. Finally, fifteen articles were eligible for quantitative review, and all of these included studies used CLSI guideline for the interpretation of inhibition zones (Fig. 1). In terms of quality assessment, the quality score of all the studies ranged from 5 to 8 points, with an average of 6.13 points, reflecting a moderate risk of bias and quality (Table 1).

**Table 1:** Quality assessment of the included studies using the Newcastle-Ottawa checklist

Author (Year)	Rating			
	Selectiondomain	Comparabilitydomain	Exposure/outcomedomain	Score
Park et al. (2018)	+ +	+ + +	+	6
Kang (2020a)	+ +	+ +	+	5
Iyori et al. (2021)	+ + +	+ + +	+ +	8
Parvathy (2018)	+ +	+ +	+ +	6
Gharajalar et al. (2023)	+ +	+ +	++	6
Naziri et al. (2023)	+ +	+ + +	+	6
AnandaChitra et al. (2016)	+ +	+ +	++	6
Lai et al. (2022)	+ +	+	+	4
Mustak et al. (2020)	+ +	+ +	+	5
Lim et al. (2020)	+ + +	+ +	+	6
Rana et al. (2022)	+ + +	+ + +	+ +	8
Putriningsih et al. (2024)	+	+ + +	+ +	6
Raja et al. (2024)	+ + +	+ +	+ +	7
Sigirci et al. (2019)	+ +	+ +	+ +	6
Kang and Hwang (2020a)	+ + +	+ +	++	7

### Prevalence of MSSP and MRSP from dog pyoderma

For the prevalence of MSSP strains isolated from dog skin infection, 14 studies were included in the meta-analysis. Across these studies, 1321 MSSP strains were identified from 2,727 *S. pseudintermedius* isolates, corresponding to a crude proportion of 48.4%. However, using a random-effects meta-analysis model, the pooled prevalence of MSSP was estimated at 27.9% (95% CI 18.8–39.3). The Q-value was 211.5 with 13 degrees of freedom and *p* < 0.001, reflecting that the true effect size differs among the included studies. This was further confirmed by the high level of heterogeneity (I^2^= 93.85%; *p* < 0.001), hence the random effects model was performed to obtain the pooled prevalence of MSSP among the samples. Publication bias was detected as both Begg’s test (*p* = 0.028) and Egger’s test (*p* < 0.001) were statistically significant.

The pooled prevalence of MRSP strains was calculated from 14 studies included in the meta-analysis. Across the 14 included studies, 1045 MRSP strains were detected from 2,721 isolates (crude proportion: 38.4%). The pooled prevalence of MRSP, estimated using a random-effects model, was 30.7% (95% CI 21.3–42.1). The Q-value was 172.1 with 13 degrees of freedom and *p* < 0.001, again, reflecting that the true effect size differs among the included studies. This was further confirmed by the high level of heterogeneity (I^2^ = 92.44%; *p* < 0.001), so the random effects model was performed to obtain the pooled prevalence of MRSP among the samples. However, no publication bias was detected as both Begg’s test (*p* = 0.125) and Egger’s test (*p* = 0.23) were not statistically significant. Random-effects meta-regression with bacterial type as a moderator showed no statistically significant difference between pooled prevalence estimates of MRSP and MSSP (slope = − 0.14, 95% CI − 1.62 to 1.34, *p* = 0.853).

### Antimicrobial resistance profile of MRSP and MSSP

**Fig. 2:**
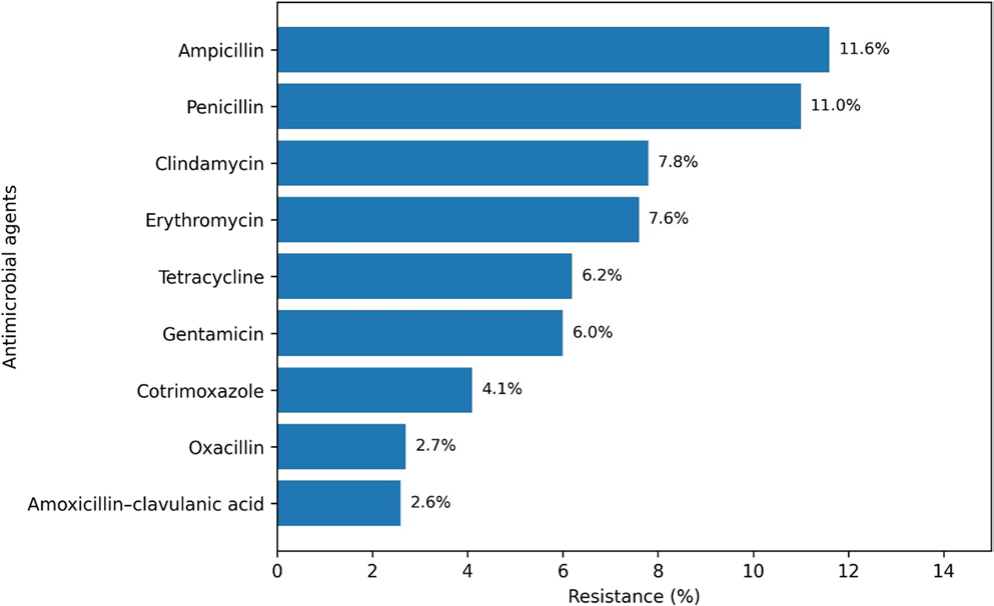
Illustrates the resistance profile of MSSP to various antimicrobials

The overall pooled estimate of the resistance of MSSP and MRSP to a specific antimicrobial agent is shown in Tables 2 and 3. The resistance of MSSP varied across the antimicrobials evaluated. The lowest resistance was observed for amoxicillin–clavulanic acid (2.6%), followed closely by oxacillin (2.7%). Cotrimoxazole showed a resistance of 4.1%. Moderate resistance levels were detected for gentamicin (6.0%) and tetracycline (6.2%). Higher resistance was observed for erythromycin (7.6%) and clindamycin (7.8%). The highest resistance was recorded for penicillin (11.0%) and ampicillin (11.6%) (Fig. 2). Overall, amoxicillin–clavulanic acid followed by oxacillin and cotrimoxazole demonstrated the lowest resistance among the antimicrobials assessed, indicating it as the most effective option against MSSP (Table 2).

**Table 2:** Meta-analysis of antimicrobial resistance of MSSP isolated from dog skin

Antimicrobial drug	Number of studies	Number of isolates	Pool estimate (%)	95% CI	*p*-value	I^2^ (%)
Doxycycline	2	15	12.7	4.1–33.4	0.002^*^	71.71
Ampicillin	3	37	11.6	3-35.3	0.005^*^	91.63
Penicillin	5	42	11	4.6–24	< 0.001^*^	84.63
Clindamycin	3	10	7.8	4.2–13.8	< 0.001^*^	0.00
Erythromycin	5	28	7.6	4.2–13.4	< 0.001^*^	57.24
Tetracycline	5	28	6.2	2.2–16.1	< 0.001^*^	81.94
Gentamicin	5	22	6	2.6–13.5	< 0.001^*^	71.17
Cotrimoxazole	5	13	4.1	1.5–10.9	< 0.001^*^	59.19
Oxacillin	5	6	2.7	1.3–5.5	< 0.001^*^	44.23
Amoxycillin-clavulanic acid	3	13	2.6	0.6–26.2	0.006^*^	85.38
Amikacin	2	1	1.4	0.3–6.9	< 0.001^*^	0.00

^*^Indicated values denote statistical significance. Values were considered statistically significant at *p* < 0.05

**Fig. 3:**
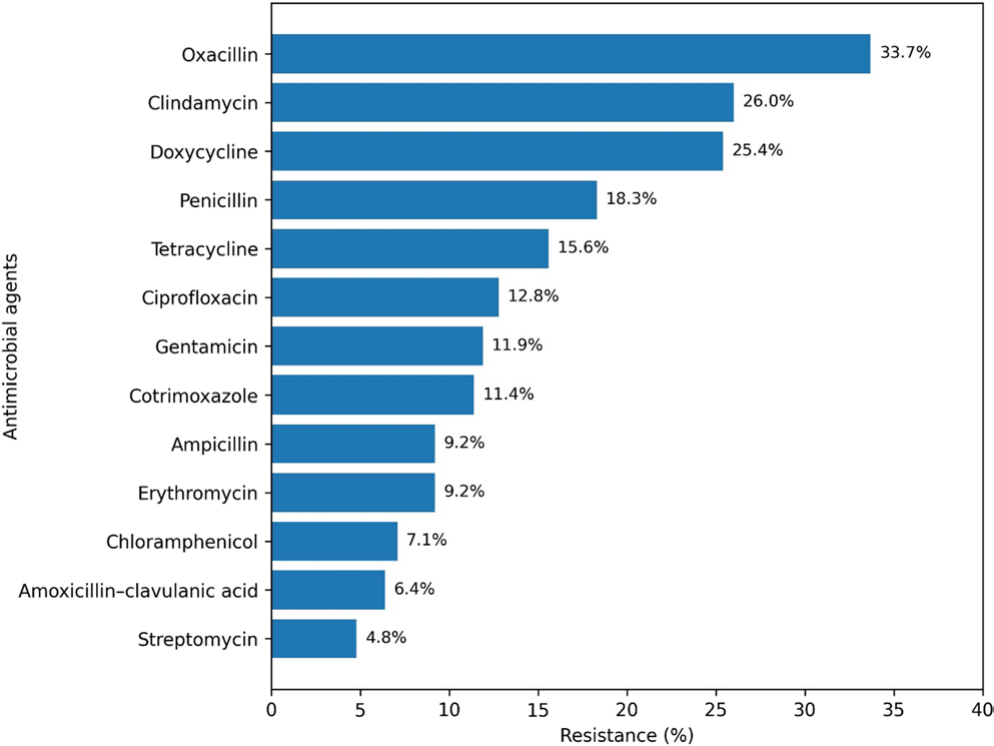
Illustrates the resistance profile of MRSP to various antimicrobials

The resistance rate among MRSP isolates showed substantial variation across the antimicrobials evaluated. The lowest resistance was observed for streptomycin (4.8%), followed by amoxicillin–clavulanic acid (6.4%) and chloramphenicol (7.1%). Resistance to erythromycin and ampicillin was identical (9.2%), while cotrimoxazole and gentamicin exhibited resistance rates of 11.4% and 11.9%, respectively. Ciprofloxacin showed a resistance rate of 12.8%, and tetracycline reached 15.6%. Higher resistance levels were detected for penicillin (18.3%), doxycycline (25.4%), and clindamycin (26.0%). The highest resistance was recorded for oxacillin (33.7%), and no studies were found for MSSP for chloramphenicol and ciprofloxacin based on the filters applied (Fig. 3). Overall, streptomycin, followed by amoxicillin-clavulanic acid and chloramphenicol, demonstrated the lowest resistance rate among the antimicrobials assessed for MRSP isolates (Table 3).

**Table 3:** Meta-analysis of antimicrobial resistance of MRSP isolated from dog skin

Antimicrobial drug	Number of studies	Number of isolates	Pool estimate (%)	95% CI	*p*-value	I^2^ (%)
Oxacillin	6	118	33.7	18.4–53.4	0.102	90.62
Clindamycin	3	42	26	9.5–54.3	0.092	88.11
Doxycycline	3	36	25.4	14-41.7	0.004^*^	68.7
Penicillin	6	83	18.3	5.7–45.2	0.024^*^	93.79
Enrofloxacin	2	17	17.4	7.5–35.3	0.001^*^	54.34
Tetracycline	6	72	15.8	4.4–43.1	0.019^*^	94
Ciprofloxacin	6	55	12.8	4.8–29.7	< 0.001^*^	90.19
Gentamicin	6	52	11.9	4-30.4	0.001^*^	89.71
Ceftriaxone	2	14	11.9	7.1–19.1	< 0.001^*^	0.00
Cotrimoxazole	6	59	11.4	3.6–30.5	0.001^*^	92.23
Ampicillin	4	28	9.2	3-24.6	< 0.001^*^	81.86
Erythromycin	6	52	9.2	2.6–27.6	0.001^*^	92.67
Chloramphenicol	3	23	7.1	0.5–52.1	0.058	92.82
Amoxycillin-clavulanic acid	4	23	6.4	2.2–17.2	< 0.001^*^	79.43
Streptomycin	3	16	4.8	0.7–25.8	0.002^*^	85.61
Amikacin	2	0	0.8	0.1–5.6	< 0.001^*^	0.00

*Indicated values denote statistical significance. Values were considered statistically significant at p < 0.05

### Prevalence of resistance genes

Among the included studies, there were reports of *mec*A gene resistance only from MRSP isolates. Therefore, for the prevalence of *mec*A in MRSP strains, 5 studies were included in the meta-analysis. Of the 5 studies, a total of 52 MRSP strains out of 389 carried *mec*A gene. The Q-value was 37.13 with 4 degrees of freedom and *p* < 0.001, reflecting that the true effect size differs among the included studies. This was further confirmed by the high level of heterogeneity (I^2^= 89.22%; *p* < 0.001), hence the random effects model was performed to obtain the pooled prevalence of resistance gene among the samples, which is 11.9% (CI: 4.4–27.8%). Publication bias was not detected as Begg’s test (*p* = 0.086) was not statistically significant.

## Discussion

This meta-analysis provides an updated quantitative overview of the prevalence and antimicrobial resistance patterns of *Staphylococcus pseudintermedius* associated with canine pyoderma. The results of pooled prevalence illustrate that the prevalence of MSSP in pyodermic dogs is 27.9%, whereas this value for MRSP isolates is 30.7%. These reports are consistent with the MSSP and MRSP isolates from dogs that were reported in another systematic review from dogs, where the pooled prevalence was reported to be 18.3% and 3.1% for MSSP and MRSP, respectively (Nasir et al. 2022). Moreover, individual clinical studies in dogs with pyoderma describe MRSP prevalences around 24–40%; for instance, Beck et al. (2012) found MRSP in 40.5% of dogs with bacterial pyoderma in Canada. These reports support the dominance of *S. pseudintermedius* in canine pyoderma, which is similar to what has been reported in Asian countries. However, there was not a significant difference between the prevalence of MSSP and MRSP reports, which may be because the strains are not different species and both reside on canine skin, meaning their colonization potential might be similar, regardless of resistance status.

Resistance profile of MRSP was broader than that of MSSP; this may be because 11.9% of MRSP isolates carried *mec*A gene. The resistance to oxacillin was considerably higher (33%) than that of MSSP isolates (2.7%), which must have come from the resistance gene *mec*A; this gene can generate β-lactamase enzymes that play a primary role in impeding the function of β-lactam antimicrobial agents (Bush and Bradford 2020). Similar reports have also been reported in Asian countries like Thailand (37.9%) (Putriningsih et al. 2024) and South Korea (100%) (Lee et al. 2018) and European countries such as Belgium (51%) (Dewulf et al. 2025) and Germany (12.2%) (Feßler et al. 2022).

Resistance to penicillin and ampicillin in MRSP isolates was 18.3% and 9.2%, respectively. In MSSP isolates, resistance rates were 11% for penicillin and 11.6% for ampicillin; these represented the highest resistance levels observed in MSSP. Generally, *Staphylococcus pseudintermedius* was susceptible to penicillinase-stable β-lactam antibiotics in the past, but since 2006, MRSP has been detected as a threat to animal health (Van Duijkeren et al. 2011). Individual studies have reported even higher resistance rates; a study in Argentina showed all 20 MRSP strains isolated from 3 different hospitals were resistant to penicillin (Gagetti et al. 2019). Furthermore, another study on dogs in Australia revealed that all strains collected from these animals were resistant to penicillin (Siak et al. 2014). Other studies in Switzerland and South Africa also found that resistance rates to ampicillin were about 50% and 57.9%, respectively (Gandolfi-Decristophoris et al. 2013; Qekwana et al. 2017). Similarly, all isolates of *S. pseudintermedius* from dogs in Korea showed resistance against ampicillin, which may be due to the ability to produce biofilm (Lee and Yang, 2020). Another reason could be using the same antimicrobial agents in both veterinary and human medicine (Iskandar et al. 2020). This resistance could be due to the presence of β-lactamase coding genes, as reported by Ruscher et al. (2009), who found all isolated MRSP strains carried the *bla*Z gene that codes for resistance against penicillin. However, it should be noted that our results are an overall resistance profile rather than individual studies, which suggest that these penicillin antimicrobial agents can still be employed, provided that a prior antimicrobial sensitivity test is performed before prescription.

Resistance to cotrimoxazole (trimethoprim-sulphamethoxazole) was slightly higher in MRSP strains (11.4%) than MSSP (4.1%). Higher resistance rates have been reported in other countries, like Italy (60%) (Bellato et al. 2022), and Turkey (Metiner et al. 2015), while lower resistance rates were reported in Iran (13.3%) (Naziri and Majlesi 2023) and India (30%) (AnandaChitra et al. 2016). These variations towards this antimicrobial agent may have come from different geographical localities, study period, the usage of the antimicrobial in that specific country and also carriage of the *mec*A gene, because it has been reported that MRSP strains carrying the *mec*A gene were significantly resistant to sulfamethoxazole/trimethoprim in dogs (Bellato et al. 2022).

The resistance towards aminoglycosides in methicillin-resistant strains showed low rates for MRSP and MSSP, with the lowest resistance towards streptomycin (4.8%) and gentamicin (6-11.9%). Two MRSP strains detected in Thailand have been reported to carry streptomycin (*aphA3*) and gentamicin (*aacA-aphD*) resistance genes, which may justify the presence of resistance to these aminoglycosides (Soimala et al. 2020). A low resistance rate was also reported from India (16.6%) against streptomycin and no resistance to gentamicin (Raja et al. 2024). On the other hand, higher resistance rates have been reported from Japan, 62.2% (Usui et al. 2025). This higher resistance in the study was reported to be due to carriage of *mec*A among the MRSP isolates. Four of the five fractions of these strains harbored at least one aminoglycoside resistance gene as well (Usui et al. 2025). These differences may be attributed to variations in the extent of aminoglycoside use across countries, which warrants further investigation. In addition, the fact that these antibacterials are widely used among humans and their dogs may have led to the exchange of resistant genes between the two species of *Staphylococcus* spp., and should not be overlooked (Frosini et al. 2020).

Resistance to tetracyclines for MRSP strains was higher from (15% to tetracycline) and (25.4% to doxycycline) than MSSP strains (6.2% to tetracycline). Similar findings have been reported by Yang et al. (2017) in Taiwan, where 55.7% MRSP isolates were resistant against tetracycline; furthermore, higher resistance levels have been reported from pet dogs with pyoderma in South Korea, where 93.4% of MRSP isolates from these dogs were tetracycline resistant (Kang and Hwang 2020b). The methicillin resistance has been reported to be significantly correlated with the resistance to tetracycline and trimethoprim (*p* = 0.033; OR = 7.1 for both antibiotics) (Han et al. 2018). Not only has this high resistance been reported in Asia, but in Europe as well; Haenni et al. (2014) reported more than 70% resistance to tobramycin, gentamicin and tetracycline in France. Furthermore, a study conducted by Scarpellini et al. (2025) in Italy reported that 97.5% of the MRSP isolates were resistant to tetracycline. Resistance to tetracyclines is frequently mediated in animal Staphylococci via the genes *tet(K)* and *tet(L)*, which code for membrane-associated efflux proteins of the major facilitator superfamily. It should be noted that the tetracycline resistance genes *tet(S)* and *tet(W)*, both coding for ribosome protective proteins, were recently identified in Staphylococci isolated from retail ground meat, and another *tet* gene, *tet(38)*, coding for a major facilitator efflux pump, was recently identified in a *Staphylococcus* isolate of unknown origin (Schwarz et al. 2018). In addition, it may be because animals have been exposed to antibiotics that make them more resistant to antibiotics, as canine hospitalization is recorded for the resistant isolates from these animals (Menandro et al. 2019).

Resistance to erythromycin was to some extent similar in both MRSP (9.2%) and MSSP (6.2%), which indicates that this macrolide can still be carefully used in cases of pyoderma. The susceptibility and/or resistance to this macrolide antimicrobial can be due to the carriage of resistance genes like *erm(A)*, *erm(C)*, and *msr(A)* encoding macrolides methyltransferases (Ruzauskas et al. 2015). The increased tolerance of microorganisms to certain antimicrobials may be attributed to frequent exposure to the same drug(s) or to lower doses of the drug. Furthermore, the use of antimicrobial agents that belong to the same category and have similar mechanisms of action against a particular organism may promote the development of greater resistance (Rana et al. 2022). Thus, the combined usage of β-lactams and fluoroquinolones as broad-spectrum antimicrobials in pets may have a significant impact on the emergence of MRSA and MRSP in dogs (Guardabassi et al. 2013).

Resistance to amoxicillin-clavulanic acid was among the lowest rates for both MSSP (2.6%) and MRSP (6.4%). Various resistance profiles have been reported to amoxycillin-clavulanic acid (AMC). In a study in Australia, no *S. pseudintermedius* isolates from healthy dogs and cats showed resistance against amoxicillin-clavulanate (Bean and Wigmore 2016). On the other hand, a retrospective study in Malaysia reported 37.9% of resistance to AMC among dogs (Haulisah et al. 2022), while *S. pseudintermedius* isolates from Turkey were reported to be 100% susceptible to this antimicrobial agent (Metiner et al. 2015). These variations have also been attributed to various clones, such as clone 71, which is reported to be resistant to beta-lactams (Paola and Luisa 2024). Moreover, this indicates that isolates from different countries may illustrate different resistance profiles and clones. As our resistance profiles are from 3 to 4 studies, the results should be interpreted with caution due to variations among studies.

Clindamycin was among the low-level resistant profile antimicrobials for MSSP (7.8%), but among the highest (26%) for MRSP isolates. Similar to resistance to other antimicrobials, one key mechanism underlying clindamycin resistance is the presence of resistance genes; notably, the *erm* gene mediates resistance to macrolides, lincosamides, and streptogramin B (MLSB) through modification of the target site on the 23 S rRNA of the ribosome, which was reported to be among most of MRSP isolates from dogs (84.2%) in Thailand (Chanchaithong and Prapasarakul 2016). The ability to produce biofilm is another way of resisting antibiotics; in a study in Iran, most of the *S. pseudintermedius* isolates produced biofilms, which have conferred resistance against clindamycin and other antimicrobials (Naziri and Majlesi 2023). Exposure to the antibiotic has also been shown to play a major role in resistance to clindamycin. Clindamycin resistance among methicillin-susceptible *S. pseudintermedius* (MSSP) isolates was significantly higher in dogs with prior antimicrobial treatment (37.7%) than in dogs without previous exposure (21.7%) (VanDamme et al. 2020).

Finally, resistance to chloramphenicol and ciprofloxacin was among the lowest (7.1%) and moderate (12.8%) for MRSP, respectively. Ciprofloxacin is a common antimicrobial agent used in veterinary medicine and is used to screen staphylococcal resistance against fluoroquinolones. Fluoroquinolone resistance in Staphylococci is largely due to mutations in the topoisomerase genes *gyrA*, *gyrB*, *grlA*, and *grlB* that resulted in amino acid substitutions in the quinolone-resistance-determining domains. In *S. pseudintermedius*, such alterations have been observed (Schwarz et al. 2018). A study on *S. pseudintermedius* in Argentina indicated that some of the isolated strains carry topoisomerase and are ciprofloxacin-resistant (Gagetti et al. 2019). In addition, findings by Loncaric et al. (2019) disclosed that MRSP isolated from companion animals, including dogs, carry *mec*A and were resistant against ciprofloxacin and enrofloxacin. According to reports, dogs are frequently found to carry multi-drug resistant (MDR) *S. pseudintermedius* following recovery from infection, and dogs that are infected with MDR strains are at a greater risk of being reinfected with *S. pseudintermedius* (Frosini et al. 2022). Higher resistance of *S. pseudinermedius* isolates from dogs has been reported to chloramphenicol in a longitudinal study in the United States, with 28% resistance rate (Calabro et al. 2024). However, nearly similar resistance rates (15.5%) have been reported from Portugal, which also carried *catp* resistance gene, a gene that codes for chloramphenicol acetyltransferase (CAT) that inactivates chloramphenicol by its acetylation (Morais et al. 2023).

It should be mentioned that the pooled prevalence of both MRSP and MSSP reported in this meta-analysis is only from studies that performed disk diffusion test on dog pyoderma and does not encompass the other types of samples and susceptibility testing methods, which is a limitation of the study and cannot represent the true prevalence of these isolates from the whole of Asia. Hence, the results that are given should only be interpreted for the disk diffusion purpose, and when samples are taken from pyodermic dogs. Similarly, the prevalence of the *mec*A gene reported in this study is restricted to the filters set (pyodermic dogs and disc diffusion test method); hence, the reported *mec*A prevalence can not represent the true prevalence of the gene in Asia. Moreover, some included studies (Parvathy 2018; Mustak et al., 2020; Lai et al., 2022) reported methicillin resistance based on *mec*A gene detection alone. Although *mecA* is a key genetic determinant of methicillin resistance, its presence does not always correlate with phenotypic expression; therefore, reliance on genotypic data alone may introduce variability in MRSP classification. Antimicrobial agents reported in only two studies (amikacin, doxycycline, ceftriaxone and enrofloxacin) were not included in the meta-analysis because pooling estimates from such a small number of studies would yield unstable and unreliable summary estimates. In addition, as we only included studies that took samples from pyoderma sites of dogs, other anatomical regions were not considered, which can be included in future studies to compare whether the anatomical sites from which the bacterium is isolated have any impact on the resistance profile or not. Finally, future studies in Asia should focus more on the resistance genes isolated from both MRSP and MSSP from dogs, as we could not find studies that addressed resistance genes other than *mec*A in dogs. Finally, another limitation of this study is that subgroup or country-level regional analyses were not performed; therefore, potential geographical differences that may influence variability in resistance profiles could not be evaluated. Future studies may incorporate additional antimicrobial testing methods to determine whether methodological differences influence reported resistance patterns. Resistance genes reported from *S. pseudintermedius* isolates can be further evaluated to better understand the distribution and prevalence of specific resistance genes among canine and feline isolates, which would provide deeper insight into resistance mechanisms and support more targeted antimicrobial stewardship strategies.

## Conclusion

This meta-analysis confirms that *S. pseudintermedius* remains the dominant bacterial agent associated with canine pyoderma, with comparable pooled prevalences of MSSP (27.9%) and MRSP (30.7%), indicating that methicillin resistance does not substantially affect colonization potential. MRSP isolates exhibited a markedly broader and more concerning antimicrobial resistance profile than MSSP, largely driven by the presence of resistance determinants such as *mec*A and other co-associated genes. Resistance to β-lactams, tetracyclines, clindamycin, and selected fluoroquinolones was consistently higher in MRSP, highlighting its multidrug-resistant nature and the growing therapeutic challenge it poses in veterinary practice. However, as the analysis was restricted to studies employing the disk diffusion method and focusing specifically on canine pyoderma cases, these findings should not be generalized to all *S. pseudintermedius* isolates circulating in Asia or to isolates derived from other infection types or testing methodologies.

Despite this, relatively low resistance rates to amoxicillin–clavulanic acid, aminoglycosides, erythromycin, and chloramphenicol suggest that certain antimicrobials may still retain clinical utility, provided that treatment is guided by antimicrobial susceptibility testing. The observed geographical variability in resistance patterns underscores the influence of antimicrobial usage practices and clonal dissemination across regions. Overall, these findings emphasize the need for prudent antimicrobial use, routine susceptibility testing, and expanded molecular surveillance, particularly in Asia, to better characterize resistance mechanisms beyond *mec*A and to inform effective control and treatment strategies for canine pyoderma. Nevertheless, the applied inclusion criteria may limit external validity, and therefore, the results should be interpreted within the defined methodological and clinical scope of this review.

## Data Availability

Datasets will be available upon a reasonable request.
